# The effect of balance training with stroboscopic glasses on postural stability and activity level in patients: a meta-analysis

**DOI:** 10.4314/ahs.v25i3.23

**Published:** 2025-09

**Authors:** Oğuzhan Bahadir Demir, Aylin Bilgin

**Affiliations:** 1 Department of Physiotherapy and Rehabilitation, Faculty of Health Sciences, Sakarya University of Applied Sciences, Sakarya, Türkiye; 2 Nursing Department, Faculty of Health Sciences, Sakarya University of Applied Sciences, Sakarya, Türkiye

**Keywords:** Activity level, chronic ankle instability, multiple sclerosis, postural stability, stroboscopic glasses

## Abstract

**Background:**

Sensory integration, particularly visual feedback, is essential for motor control and balance. Stroboscopic glasses, which intermittently restrict visual input, have emerged as a promising tool for rehabilitation and performance enhancement.

**Objective:**

This systematic review and meta-analysis evaluated the impact of balance training with stroboscopic glasses on postural stability and activity levels in patients with chronic ankle instability or multiple sclerosis.

**Methods:**

A systematic search of Science Direct, Web of Science, and PubMed was conducted up to September 2024 using relevant keywords. Randomized controlled trials published in English were included, excluding studies with incomplete data or abstract-only publications. From 5,691 screened records, five studies met the criteria, involving predominantly male patients with chronic ankle instability or multiple sclerosis.

**Result:**

Balance training with stroboscopic glasses significantly improved postural stability (Z = 4.83, p < 0.001), while changes in activity scores were not statistically significant. Most studies demonstrated low risk of bias and strong methodological quality.

**Conclusion:**

Stroboscopic glasses appear effective in enhancing postural stability, though their impact on activity levels remains unclear. Larger, diverse samples and comparative studies with other training methods are needed to validate these findings and explore long-term benefits.

## Introduction

Stroboscopic glasses have emerged as an innovative tool for the rehabilitation of visual perception and motor skills in recent years. These glasses aim to optimize individuals' attention, balance, coordination, and reaction times by intermittently restricting visual feedback. The stroboscopic effect is a visual phenomenon in which continuous movements (e.g., rotation or other cyclical motions) are represented through brief, momentary samples. This approach has been integrated into training programs related to popular sports activities among young adults^1^. The fundamental rationale behind this training is that the interruption of visual information forces individuals to reduce their dependence on environmental visual feedback. This training method encourages users treduce their dependence on visual information and enhance their motor skills without eliminating visual input. Previous research has shown that stroboscopic training positively affects predictive timing^2^, visual cognition^3^, as well as visual attention and information encoding^4^.

Balance training has been shown in the literature to improve functional outcomes in individuals with chronic ankle instability^5^. Increasing the difficulty level in balance training allows the sensorimotor system to adapt to more challenging tasks^5^. However, it has been reported that individuals with chronic ankle instability are more dependent on visual information in single-leg balance assessments compared to their uninjured control counterparts^6^,^7^. Stroboscopic training reduces visual input by repeatedly turning it on and off, rather than eliminating it. The stroboscopic glasses create a visual disruption by making visual stimuli discrete, which can pose challenges to visual cognition^8^. This mechanism plays a crucial role in providing visual feedback for postural control in individuals with somatosensory deficiencies and can be beneficial in rehabilitation programs aimed at improving motor control^9^,^10^. The potential of stroboscopic training to facilitate Anterior Cruciate Ligament (ACL) rehabilitation and reduce the risk of re-injury has also been emphasized^11^.

After ACL repair, reliance on visual input can hinder postural balance during multitasking. Static postural stability assessments reveal deficiencies in proprioceptive and visual integration, which may persist for years post-surgery, highlighting long-term challenges in recovering balance control following musculoskeletal injuries^12^-^14^. Given these known sensory deficiencies, it is essential for clinics to utilize postural stability assessments during the rehabilitation and return-to-sport process to evaluate the status of sensory recovery in individuals who have undergone ACL repair. Previous studies have attempted to evaluate sensory reweighting by using stroboscopic glasses during static and dynamic tasks^15^,^16^.

The use of stroboscopic glasses in studies aimed at improving balance is a highly relevant topic, and reviewing these studies is essential to understand their overall effects and to contribute to the literature. Therefore, the primary aim of this meta-analysis is to examine the impact of balance training with stroboscopic glasses on postural stability and activity levels. In addition, this systematic review and meta-analysis also aimed to provide information about the methodological quality and risk of bias of the studies on balance training with stroboscopic glasses.

## Methods

### Study design

This study followed the Preferred Reporting Items for Systematic Reviews and Meta-Analyses (PRISMA 2020) guidelines^17^, which provide a framework for conducting systematic reviews and meta-analyses.

### Inclusion and exclusion criteria

This meta-analysis incorporated studies involving patients with diagnoses of multiple sclerosis or chronic ankle instability, where balance training interventions conducted using stroboscopic glasses were implemented to assess postural control and activity levels. Only randomized controlled studies published in English were eligible for inclusion. Studies that appeared solely as abstracts or lacked the mean (M) and standard deviation (SD) values necessary for effect size calculations were excluded from the analysis. (2) Gray literature sources such as research not published in peer-reviewed journals and not overseen by commercial publishers; (3) theses, expert opinions, letters, and conference proceedings; and (4) studies that have not been formally published. Gray literature sources, such as studies not published in peer-reviewed journals and not controlled by commercial publishing organizations, along with theses, expert opinions, letters, and conference proceedings, were excluded from this meta-analysis.

### Search strategy

Data collection involved searching online databases up to September 2024, including Science Direct, Web of Science, and PubMed. The search was conducted using the following keywords: 1) “stroboscopic” OR “strobe glasses” and 2) terms like “patients” OR “hospitalized” OR “disease” OR “illness.” In the initial phase, two authors conducted independent literature searches using the same set of keywords, without input from other individuals.

### Data Extraction

All studies gathered in the Endnote X20 (Clarivate Analytics, Philadelphia, PA, USA) reference management software was reviewed by the authors. After removing duplicates, the titles and abstracts of the remaining studies were screened by two investigators according to the inclusion criteria. The authors then independently evaluated the full-text articles based on the predefined inclusion and exclusion criteria. To ensure a detailed and consistent assessment, the authors created a standardized form capturing details such as the first author's name, publication date, inclusion criteria, sample size, age, gender, weight.

### Evidence level and Methodological quality

The levels of evidence followed the Joanna Briggs Institute hierarchy^18^. Level 1 evidence, divided into four subgroups, included systematic reviews and randomized controlled trials (RCTs). Level 1a covered systematic reviews of RCTs, 1b included reviews of RCTs and other studies, 1c referred to well-designed RCTs, and 1d to poorly designed RCTs. Methodological quality was assessed using the Quality Assessment Tool for Quantitative Studies, evaluating study design, selection bias, blinding, confounding variables, data collection methods, and withdrawals. Each item was rated as “strong,” “moderate,” or “weak,” with a global quality rating assigned. Studies without weak ratings were strong, one weak rating was moderate, and two or more were poor^19^. The quality of the included studies was assessed independently by two authors, with consensus reached through discussion.

### Risk of bias assessment

The risk of bias in the selected trials was evaluated using the revised Cochrane risk of bias tool for randomized trials (RoB 2). Each author independently assessed all included studies across five domains: (1) randomization process, (2) deviations from the intended interventions, (3) missing data, (4) outcome evaluation, and (5) selection of reported results. For each domain, the risk of bias was classified as low, moderate concern, or high. A study was classified as having a low risk of bias if all domains were rated as low risk, moderate if at least one domain raised concerns, and high risk if any domain was rated as high risk or if multiple domains were rated as moderate concern^20^.

### Data synthesis

Review Manager (RevMan, V.5.4.; The Nordic Cochrane Centre, The Cochrane Collaboration, Copenhagen) was used for the statistical assessment of the studies utilized. For the assessment of diverseness in the studies, the Chi-square statistic and the I2 statistic were employed. In analyses with more than ‘I2’ 50% and with p-value less than 0.05, the model performed the quantitative analysis using random effect models^21^. Mean difference (MD) and 95% confidence intervals (CI) were used in standardizing study outcomes. The average of all the effects took place in the calculation of the overall effect. Data analysis was illustrated in a forest plot. An asymmetrical funnel plot represents a potential publication bias. The Begg regression test was applied to test the asymmetrical funnel plot^22^. P < 0.05 was considered statistically significant.

## Results

### Study Selection

A total of 5,691 records were screened. Prior to screening, 4,561 were eliminated as duplicates, 745 were review articles only, and 385 were studies that did not contain patient data. As a result, 81 records were scanned for evaluation; of these, 74 were excluded for not meeting the inclusion criteria, and 2 reports were excluded due to having a non-randomized design. Ultimately, 5 studies that met the inclusion criteria were included in the meta-analysis.

### Study Characteristics

Two of the studies were conducted in the USA, while one was carried out in Turkey, another in Israel, and the final one in Spain. The total sample sizes in the studies ranged from 26^23^ to 52^24^ participants, with a combined sample of 204 included in the meta-analysis. The patient populations in the studies included four with a diagnosis of Chronic Ankle Instability and one with multiple sclerosis^24^. The average age of participants ranged from 20 to 29.67 years, and most participants across all studies were male. Baseline FAAM-ADL scores for participants in the intervention group ranged from 59.84 to 86.7, compared to scores of 65.05 to 87.7 in the control group. Baseline FAAM-sport scores ranged from 69.17 to 79.92 for the intervention group and from 67.29 to 80.73 for the control group (Table 1).

**Table 1:** Characteristics of Reviewed Studies

Study (Years)/Country	Inclusion criteria	Sample	Age (Mean±SD)	Gender (Percent)	Mass (kg)	Height (cm)	FAAM-ADL	FAAM-Sports
**Kim et al. (2021) Spain**	- a prior ankle sprain at least 6 months prior to the study's start - a score of 24 or lower on the CAIT to indicate current ankle joint instability. - no history of other musculoskeletal injuries in the lower extremities - mental and physical autonomy to engage in the intervention.	Intervention: 24 Control: 24	Intervention: 27.38 (7.38) Control: 29.67 (9.40)	Intervention: Male: 17 (70.83%) Female: 7 (29.17%) Control: Male: 13 (54.17%) Female: 11 (45.83%)	Intervention: 71.94 (9.89) Control: 69.38 (9.18)	Intervention: 170.63 (8.79) Control: 170.50 (9.75)	Intervention: 74.04 (8.17) Control: 72.50 (9.32)	Intervention: 69.17 (6.19) Control: 67.29 (6.25)
**Lee et al. (2022) USA**	- A history of at least one significant ankle sprain occurring more than 3 months prior to data collection. - A history of two giving way episodes within the past six months. - A score of less than 90% on the FAAM-ADL. - A score of less than 80% on the FAAM-Sports. - At least 5 “yes” answers on the AII, including question 1 and 4 additional questions. - A history of engaging in physical activity at least 3 days a week for a total of 90 minutes per week in the previous 3 months.	Intervention: 14 Control: 14	Intervention: 21 (3) Control: 22 (2)	Intervention: Male: 6 (70.83%) Female: 8 (29.17%) Control: Male: 8 (54.17%) Female: 6 (45.83%)	Intervention: 72 (11) Control: 71 (12)	Intervention: 175.5 (8.0) Control: 173.5 (8.3)	Intervention: 86.7 (8.7) Control: 87.7 (8.7)	Intervention: 72.5 (8.7) Control: 72.9 (8.7)
**Lee et al. (2024) USA**	Patients were included according to the guidelines of the International Ankle Consortium.	Intervention: 25 Control: 25	Intervention: 22 (3) Control: 21 (2)	Intervention: Male: 13 (70.83%) Female: 12 (29.17%) Control: Male: 12 (54.17%) Female: 13 (45.83%)	Intervention: 71.8 (12.2) Control: 71.1 (13.5)	Intervention: 174.7 (8.2) Control: 173.1 (8.3)	Intervention: 85.0 (8.3) Control: 85.8 (6.4)	Intervention: 70.3 (9.3) Control: 68.6 (13.4)
**Shalmoni & Kalron (2020) Israel**	- diagnosis of definite MS according to the revised McDonald criteria 2010 - Between 25 and 55 years of age - Expanded Disability Status Scale score ranging from 2.0 to 5.5. - ability to understand and execute simple instructions	Intervention: 26 Control: 26	47.9 (9.1)	Male: 16 (70.83%) Female: 10 (29.17%)	67.1 (16.0)	160 (1.0)	-	-
**Uzlaşır et al. (2021) Turkey**	- Between 18 and 25 years of age - A history of moderate to severe unilateral ankle sprain (resulting in 8 days of sports time loss) associated with inflammatory symptoms, pain, and swelling within the last 5 years, but at least 1 year before enrollment. - Experiencing at least 2 episodes of the ankle giving way in the 6 months prior to enrollment. - A score of 11 or higher on the IDFAI - A score of less than 90% on the FAAM-ADL - A score of less than 80% on the FAAM-Sports	Intervention: 13 Control: 13	Intervention: 20.23 (0.39) Control: 20.46 (0.51)	Intervention: Male: 6 (70.83%) Female: 7 (29.17%) Control: Male: 7 (54.17%) Female: 6 (45.83%)	Intervention: 66.06 (2.40) Control: 63.19 (3.39)	Intervention: 172 (0.2) Control: 168 (0.2)	Intervention: 59.84 (4.71) Control: 65.05 (4.33)	Intervention: 79.92 (2.82) Control: 80.73 (2.00)

**Abbreviations:** AII: Ankle Instability Instrument, ACL: Anterior cruciate ligament, CAIT: the Cumberland Ankle Instability Tool, FAAM ADL: Foot and Ankle Ability Measure for Activities of Daily Living, FAAM Sports: Foot and Ankle Ability Measure for Sports, IDFAI: Identification of Functional Ankle Instability Questionnaire, MS: Multiple Sclerosis

### Intervention

The balance training content mainly included single-limb stance, throwing, and catching tasks, single-leg deadlifts, and single-limb hops for stabilization. One study included a ball-catching exercise. The duration of each exercise session was 15-20 minutes. The exercises were applied for a minimum of 4 weeks and a maximum of 6 weeks across the studies. The total number of exercise sessions varied between 12 and 18. The control group either underwent balance training without glasses or received no intervention at all. While the source of the stroboscopic glasses was consistent across four studies, it differed in one study^24^. In the studies that conducted balance training with stroboscopic glasses, the instructors included certified athletic trainers in two studies and an expert physiotherapist in one study; however, two studies^23^,^24^ did not report information about the instructors (Table 2).

**Table 2:** Interventions of Reviewed Studies

Study (Years)/Country	Study design	Intervention	Control	Stroboscopic glasses	Intervention educator	Scales	Results
**Kim et al. (2021) Spain**	Randomized controlled trial	This intervention consisted of a supervised multimodal exercise protocol addressing different aspects of balance, including static and dynamic tasks in the injured ankle. The training program consisted of 6 exercises of increasing difficulty depending on the patient's implementation, supervised by a specialist physiotherapist. The exercises were performed in a 20-min circuit format and all participants completed 18 training sessions divided over 6 weeks. The exercises included Single-limb stance, Throwing, and catching task., single-leg dead lift, and single-limb hops for stabilization.	Participants in the control group received no intervention.	Stroboscopic eyewear (Senaptec, Beaverton, OR)	Expert physiotherapist	Balance test FAAM-ADL FAAM-sport CAIT	Better scores in stroboscopic training groups in all outcome measures were observed in comparison with the control group with moderate to large effect sizes.
**Lee et al. (2022) USA**	Randomized controlled trial	As an intervention, a 4-week dynamic balance training program was implemented to improve postural control in patients with CAI. Participants attended a total of 12 supervised 20-minute sessions, three times a week. The stroboscopic group wore stroboscopic glasses only during the sessions. The balance training included various dynamic balance activities that challenged single-leg stance, with the difficulty levels progressively increased. Activities included stabilization jumps and single-leg balance tasks.	Participants received only the rehabilitation program without stroboscopic glasses.	Stroboscopic eyewear (Senaptec, Beaverton, OR)	A certified athletic trainer	Single leg hop stabilization test Balance test FAAM-ADL FAAM-sport CAIT	The strobe group showed a higher pretest-posttest difference in velocity in the medial-lateral direction and vertical stability index under SV compared with the control group.
**Lee et al. (2024) USA**	Randomized controlled trial	A 4-week dynamic balance training program, previously used for CAI patients, was implemented, focusing on hopping and single-leg balance key components for enhancing spontaneous movement strategies and postural control. As participants advanced, environmental challenges were introduced to gradually increase exercise difficulty. The strobe group wore stroboscopic glasses during training, with opacity adjusted by increasing the glasses' frequency as exercises became more challenging. Participants in this group adjusted the glasses for optimal fit. All participants completed 12 sessions, each lasting 20 minutes, over 4 weeks, training three times per week.	Participants received only the rehabilitation program without stroboscopic glasses.	A 3 Stroboscopic eyewear (Senaptec, Beaverton, OR)	A certified athletic trainer	FAAM-ADL FAAM-sport CAIT	Patients of the strobe group demonstrated changes in neuromechanics, including increased ankle-dorsiflexion and eversion angles and tibialis anterior and peroneus longus activation, during a single-legged drop landing
**Shalmoni & Kalron (2020) Israel**	Randomized crossover study	The exercises included various drills aimed at improving the participant's motor skills. First, during the ball catch drill, the therapist and participant threw the ball back and forth from different locations and at varying speeds. The wall ball drill involved the participant standing a few meters away from the wall while attempting to catch the ball thrown by the therapist. In the head turn and catch drill, the participant stood with their back to the therapist and glanced over their right shoulder to see the ball being thrown, then turned their head to catch it. Finally, the turn and catch drill started with the participant facing away from the therapist, who called out “ball” while throwing it, prompting the participant to turn and attempt to catch it. These exercises aimed to enhance the participant's balance and reaction skills. SVT session was performed wearing stroboscopic glasses.	Participants received only the rehabilitation program without stroboscopic glasses.	Vision up Strobe battery powered glasses (Appreciate Co., Ltd., Kyoto, Japan)	-	the center of pressure (CoP) path length (cm) postural sway (cm/s2).	No differences between pre–post measurements were noted for gait and balance.
**Uzlaşır et al. (2021) Turkey**	Randomized controlled trial	Single leg jumping exercises were performed on the injured leg. These exercises have been previously described and shown to be effective for improving balance ability in chronic ankle instability (CAI). Each session began with a 5-minute warm-up that included jogging and painless Achilles tendon stretches (progressing from 3 to 7 repetitions). On average, each session was completed in 20 minutes. The exercise program included the following: Single-leg Hop to Stabilization, Hop to Stabilization and Reach, Unanticipated Hop to Stabilization, Single-leg balance. Patients performed the exercises using stroboscopic glasses.	Participants received only the rehabilitation program without stroboscopic glasses.	Stroboscopic eyewear (Senaptec, Beaverton, OR)	-	Balance velocity	Balance velocity was significantly higher in the strobe group.

**CAI:** chronic ankle instability, CAIT: the Cumberland Ankle Instability Tool, DPSI: dynamic postural stability index, EO: eyes open, FAAM ADL: Foot and Ankle Ability Measure for Activities of Daily Living, FAAM Sports: Foot and Ankle Ability Measure for Sports, SV: stroboscopic vision

### Risk of Bias

**Figure 1:**
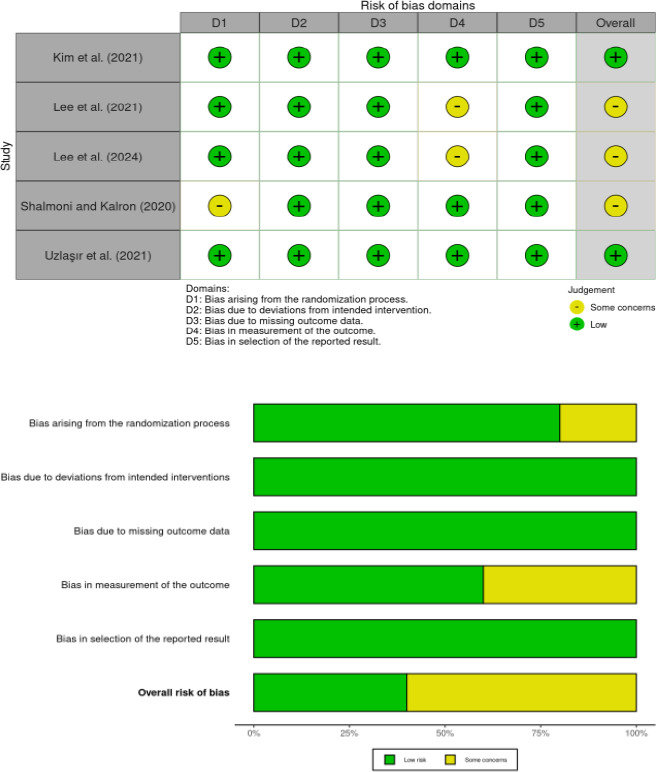
Risk of bias assessment

Four of included studies (80%) reported randomization and provided details regarding randomization, but a study (20%) indicated that a crossover method was employed. All studies were identified as having low of bias due to deviations from intended interventions and missing outcome data. Three studies (60%) had a low bias in outcome measurement due to blind the researchers who collected and evaluated the data. However, two studies (40%) had some risk of bias due to not reporting it. The risk of bias in selecting the reported outcome was low in all studies (100%). Two studies (40%) were determined to have a low overall risk of bias (Figure 1). The Begg regression test revealed no publication bias in the included studies (intercept = 0.800, P = 0.083).

### Quality Appraisal

The evidence level of our studies was level 1c (indicating a well-designed RCT), while one study was level 1d (indicating a poorly designed RCT). The methodological quality of the five included studies was evaluated, with three studies rated as “strong” overall, while two studies were rated as “moderate.” In all included studies, participants were provided with a detailed explanation related to the study, which minimized selection bias. The assessment of study design indicated that all studies had strong quality. It was reported and documented that confounding factors, such as age and gender, that could affect study outcomes were limited in all studies. Additionally, in three of the studies, blinding of data collectors was reported. Data in all studies were collected and scored using valid and reliable scales. Withdrawals and dropouts were presented in flow diagrams for all studies (Table 3).

**Table 3:** The Methodological Qualities and Evidence Level of Studies

	QUALITY ASSESSMENT TOOL FOR QUANTITATIVE STUDIES							
Study	Selection Bias	Study Design	Confounders	Blinding	Data Collection Method	Withdrawals and Dropouts	Global Rating	Evidence Level
**Kim et al. (2021)**	Strong	Strong	Strong	Strong	Strong	Strong	Strong	Level 1c
**Lee et al. (2022)**	Strong	Strong	Strong	Weak	Strong	Strong	Moderate	Level 1c
**Lee et al. (2024)**	Strong	Strong	Strong	Weak	Strong	Strong	Moderate	Level 1c
**Shalmoni & Kalron (2020)**	Strong	Strong	Strong	Strong	Strong	Strong	Strong	Level 1d
**Uzlaşır et al. (2021)**	Strong	Strong	Strong	Strong	Strong	Strong	Strong	Level 1c

### Overall outcomes

**Figure 2:**
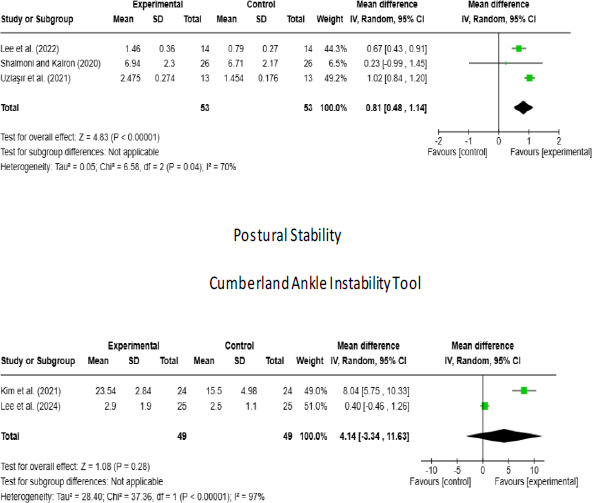
Forest plot of the effect of stroboscopic glasses intervention on Cumberland Ankle Instability Tool and Postural Stability in patients

There are a total of 3 studies 23-25 evaluating the postural stability level, with a combined sample size of 106 (Intervention: 53, Control: 53) across these studies. An examination of heterogeneity between studies revealed a Chi2-value of 6.58 (p=0.04) and an I^2^ value of 70%, indicating high heterogeneity among the studies. Therefore, a random effects model was used to determine the overall effect. According to the random effects model, the mean overall effect was estimated to be 0.81, with a confidence interval ranging from 0.48 to 1.14. The overall effect obtained from the studies is statistically significant in favor of the intervention (Z = 4.83, p < 0.001) (Figure 2).

There are a total of 2 studies 26, 27 at Level 1c evidence evaluating the CAIT level, with a combined sample size of 98 (Intervention: 49, Control: 49) across these studies. An examination of heterogeneity between studies revealed a Chi2-value of 37.36 (p < 0.001) and an P value of 97%, indicating high heterogeneity among the studies. According to the random effects model, the mean overall effect was estimated to be 4.14, with a confidence interval ranging from -3.34 to 11.63. The overall effect obtained indicates that the intervention caused an increase in CAIT scores in favor of the intervention group; however, this increase was not statistically significant (Z = 1.08, p = 0.28) (Figure 2).

**Figure 3:**
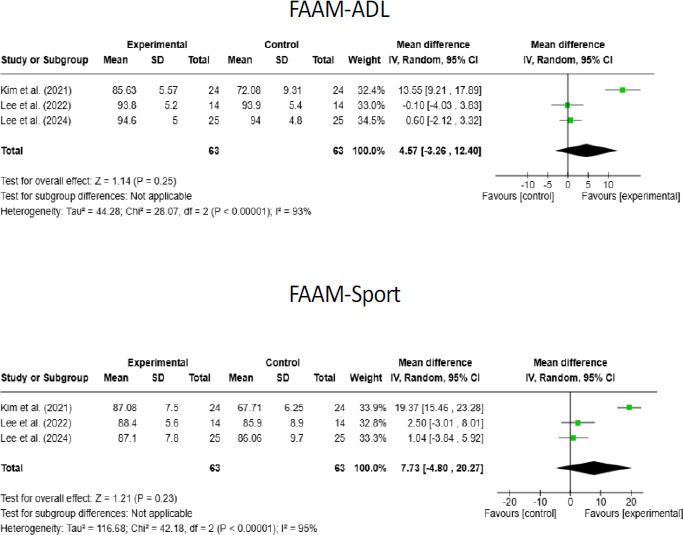
Forest plot of the effect of stroboscopic glasses intervention on FAAM-ADL and FAAM-Sport in patients

There are a total of 3 studies 25-27 at Level 1c evidence evaluating the FAAM-ADL level, with a combined sample size of 126 (Intervention: 63, Control: 63) across these studies. An examination of heterogeneity between studies revealed a Chi2-value of 28.07 (p < 0.001) and an I^2^ value of 93%, indicating high heterogeneity among the studies. According to the random effects model, the mean overall effect was estimated to be 4.57, with a confidence interval ranging from -3.26 to 12.40. The overall effect obtained indicates that the intervention caused an increase in FAAM-ADL scores in favor of the intervention group; however, this increase was not statistically significant (Z = 1.14, p = 0.25) (Figure 3).

There are a total of 3 studies 25-27 at Level 1c evidence evaluating the FAAM-Sport level, with a combined sample size of 126 (Intervention: 63, Control: 63) across these studies. An examination of heterogeneity between studies revealed a Chi2-value of 42.18 (p < 0.001) and an I2 value of 95%, indicating high heterogeneity among the studies. According to the random effects model, the mean overall effect was estimated to be 7.73, with a confidence interval ranging from -4.80 to 20.27. The overall effect obtained indicates that the intervention caused an increase in FAAM-Sport scores in favor of the intervention group; however, this increase was not statistically significant (Z = 1.21, p = 0.23) (Figure 3).

## Discussion

This systematic review and meta-analysis demonstrate that the intervention has a significant positive effect on postural stability among participants. This finding reinforces the hypothesis that targeted interventions can enhance balance and stability. Studies have shown that structured balance training can lead to significant improvements in postural stability, particularly in populations at risk for falls^28^,^29^. Maki et al. (1994)^36^ highlighted that specific balance exercises could reduce fall risk in older adults by enhancing both static and dynamic postural control, while Sherrington et al. (2017)^37^ found that balance training notably improved stability among elderly individuals. Lesinski et al. (2015) showed that balance training had long-term positive effects on muscle strength and coordination, which are critical for maintaining postural stability. The effectiveness of interventions using stroboscopic glasses has been documented in enhancing sensory integration and dynamic balance by challenging the visual and vestibular systems in a controlled manner.^1^ Ellisn et al. (2020)^30^ reported that stroboscopic training can improve hand-eye coordination and reaction time, while Appelbaum et al. (2012)^4^ noted improvements in cognitive flexibility and spatial awareness. These cognitive and perceptual improvements may indirectly contribute to better balance and postural control in patients. Stroboscopic glasses alter participants' visual perception, which enhances their ability to maintain balance.

This meta-analysis reported that the intervention had a positive effect on FAAM-ADL and FAAM Sport scores, but no statistically significant results were obtained. The use of stroboscopic glasses may create positive effects on balance and coordination, which can lead to better performance in daily living activities^1^,^31^. The use of stroboscopic glasses is considered part of strategies aimed at improving balance, particularly for individuals at high risk of falls. For these individuals, maintaining balance and conducting daily activities more safely can enhance their overall quality of life^29^,^32^. Despite the positive effect observed in this meta-analysis, the absence of statistical significance may be related to the current sample size or study design. Additionally, heterogeneity in assessment tools across studies and inconsistencies in intervention duration may have influenced the results. Differences in how functional outcomes were measured could have affected the comparability of findings, thereby diluting potential statistical effects. It is known that a specific intervention's effect often requires sufficient power to achieve statistical significance. Button et al. (2013)^33^ discussed how underpowered studies often miss significant results, suggesting that larger, well-powered studies are essential to accurately gauge intervention effectiveness. Studies with a larger sample size or participants with different demographic characteristics may better elucidate the effectiveness of the intervention.

The findings obtained from the meta-analysis indicate that interventions involving stroboscopic glasses resulted in an increase in the CAIT scores for the intervention group; however, this increase is not statistically significant. Research specifically targeting individuals with chronic ankle instability further supports the positive influence of balance training on CAIT scores. Molla-Casanova et al. (2021)^34^ found that consistent balance training programs improved stability and functional performance in patients with ankle instability. Fang et al. (2024)^35^ similarly observed that ankle-specific balance exercises could reduce symptoms of instability by improving proprioception and neuromuscular control. Cruz-diaz et al. (2015)^36^ extended these findings, showing that tailored balance programs not only enhanced CAIT scores but also mitigated recurrent ankle injuries by improving joint stabilization mechanisms. A systematic review on the impact of exercise on balance and postural control in patients with ankle instability reported improvements in CAIT scores for these patients^37^. Previous research has indicated that balance training has positive effects on CAIT scores in individuals with chronic ankle instability^34^,^36^. Guo et al. (2024)^38^ highlighted that differences in protocol duration, intensity, and exercise type can impact the efficacy of balance training interventions. Likewise, the variability in intervention length, frequency, and participant adherence in the included studies may have contributed to the lack of statistical significance. Moreover, the use of different outcome measures or scoring thresholds could have introdced measurement bias, further complicating the ability to detect meaningful changes^39^.

### Limitations

This meta-analysis has certain limitations. A large proportion of the included studies primarily involved male participants, which may limit the generalizability of the findings to females. Additionally, small sample sizes and variations in intervention protocols and outcome measures across studies may have contributed to heterogeneity and reduced statistical power. The exclusion of gray literature and unpublished studies may also have introduced publication bias.

## Conclusions

This systematic review and meta-analysis show that balance training with stroboscopic glasses significantly improves postural stability in patients. While activity scores improved with the intervention, these changes were not statistically significant. The findings indicate that stroboscopic glasses could be a valuable tool in balance training, but further research with larger and more diverse samples is necessary to confirm their effectiveness and applicability. Long-term studies are needed to assess whether these improvements persist over time. Comparing stroboscopic glasses with other balance training methods could highlight their relative advantages and drawbacks. Additionally, evaluating participants' experiences and satisfaction could provide insights into the acceptability and motivational impact of this approach. More comprehensive research addressing these aspects could better define the role of stroboscopic glasses in balance training and enhance intervention strategies.

## Fundings

The authors received no financial support for the research, authorship, and/or publication of this article.
